# Supplementary material for: A Machine Learning Model for the Proteome-Wide Prediction of Lipid-Interacting Proteins
Source: J Chem Inf Model. 2025 Sep 4;65(18):9623–38. doi: 10.1021/acs.jcim.5c01076 (PMC12458687; doi:10.1021/acs.jcim.5c01076)
Supplement: Supplementary file 1 [file ci5c01076_si_001.pdf]

Supporting Information for

A machine learning model for the proteome-wide prediction of lipid-interacting proteins

Jonathan Chiu-Chun Chou,[1] Poulami Chatterjee,[1] Cassandra M. Decosto,[1,2] Laura M. K. Dassama[1,2,3*]

[1]Department of Chemistry, Stanford University, Stanford, CA 94305

[2]Sarafan ChEM-H Institute, Stanford University, Stanford, CA 94305

[3]Department of Microbiology and Immunology, Stanford School of Medicine, Stanford, CA 94305

Supporting figures

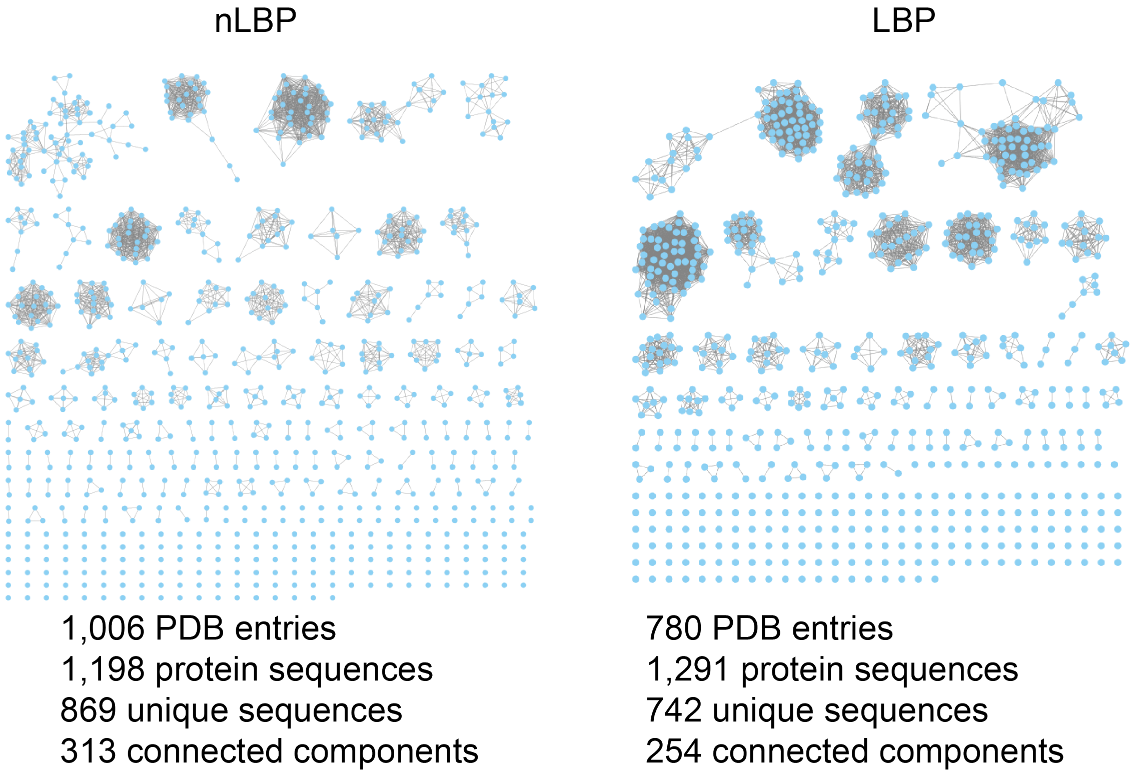

Fig. S1. Protein sequence similarity networks of non-lipid binding protein (nLBP) and lipid binding protein (LBP) training dataset. Each node represents one unique sequence and the edge represents the connection that has a E-value of 10^{-5}. Proteins with high sequence similarity will cluster together.

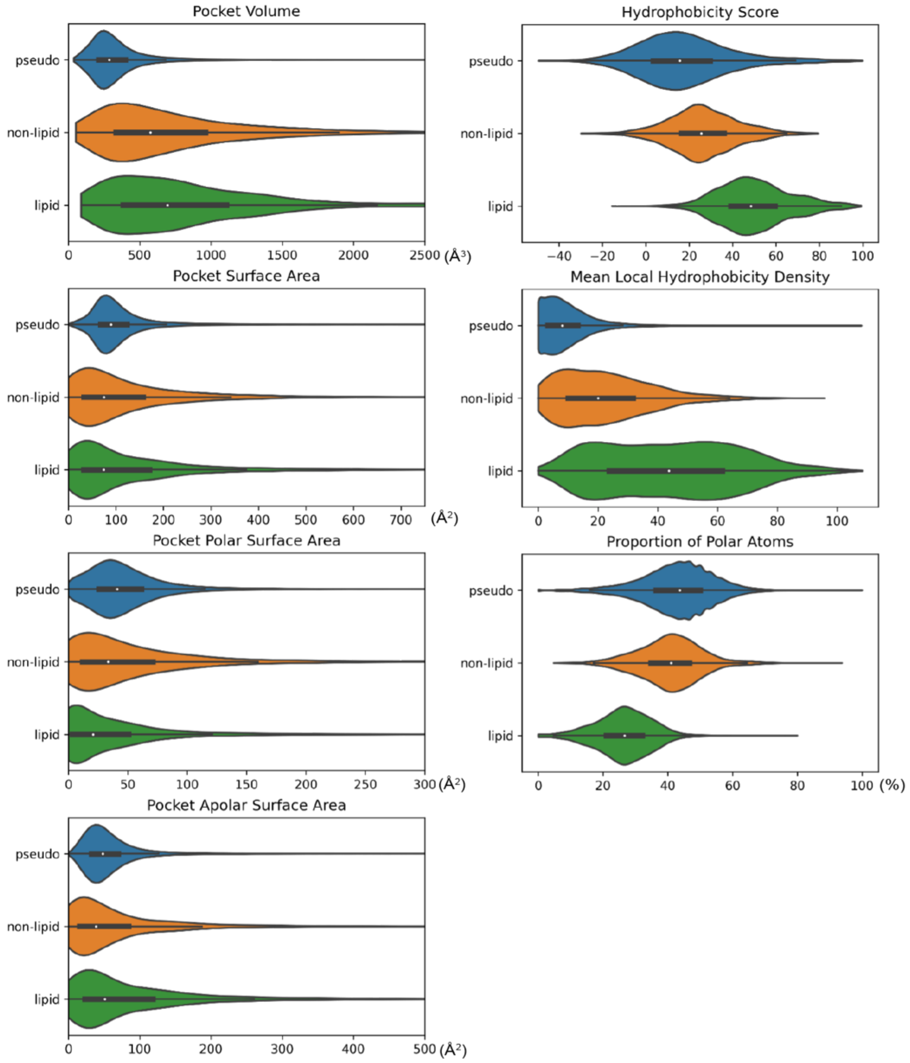

Fig. S2. Violin plots showing representative pocket descriptors of LBPs, nLBPs, and PPs. Hydrophobicity score is based on an arbitrary scale suggested by Monera et al.(1), where glycine is defined as 0 and phenylalanine as 100.

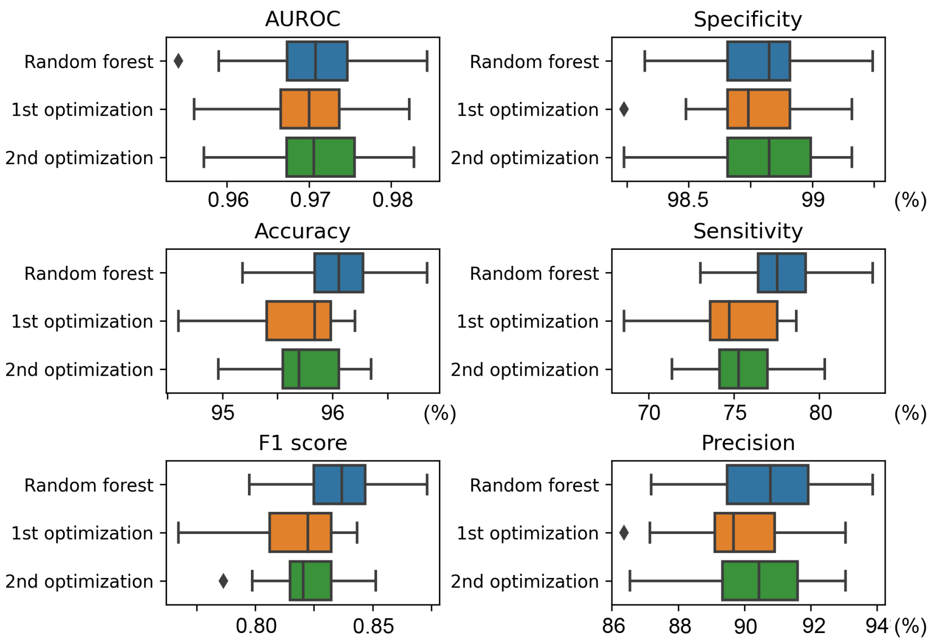

Fig. S3. Hyperparameters optimization for the classifier model. The performance was assessed with 25 random seedlings. The boxes were plotted from first quartile to third quartile, while the whiskers extend to demonstrate the whole range of the data with the exception of outliers.

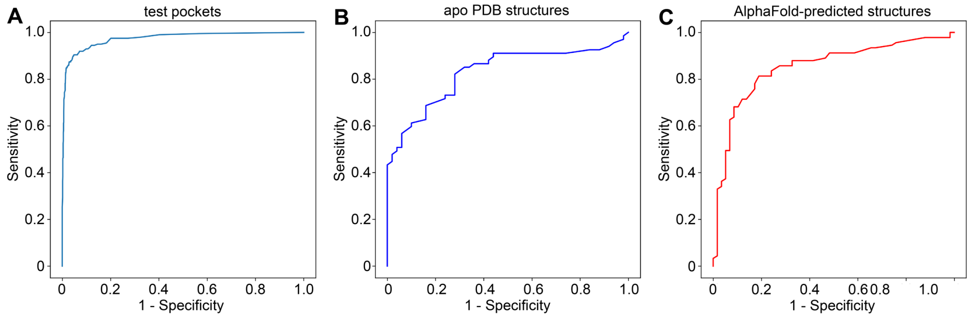

Fig. S4. Receiver operating curves for (A) the test dataset, (B) the apo PDB dataset, and (C) the AlphaFold dataset. The curves were plotted as the sensitivity vs (1 – specificity) at different thresholds.

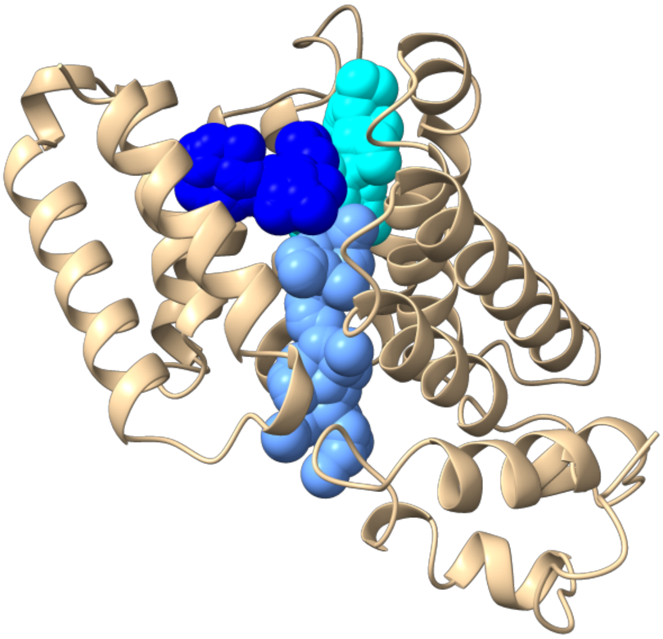

Fig. S5. Prediction of pockets within BstC (PDB 7T1S) using fpocket. AlphaFold model is colored in wheat and the predicted pockets is colored in various shades of blue to indicate separate the pockets predicted by fpocket.

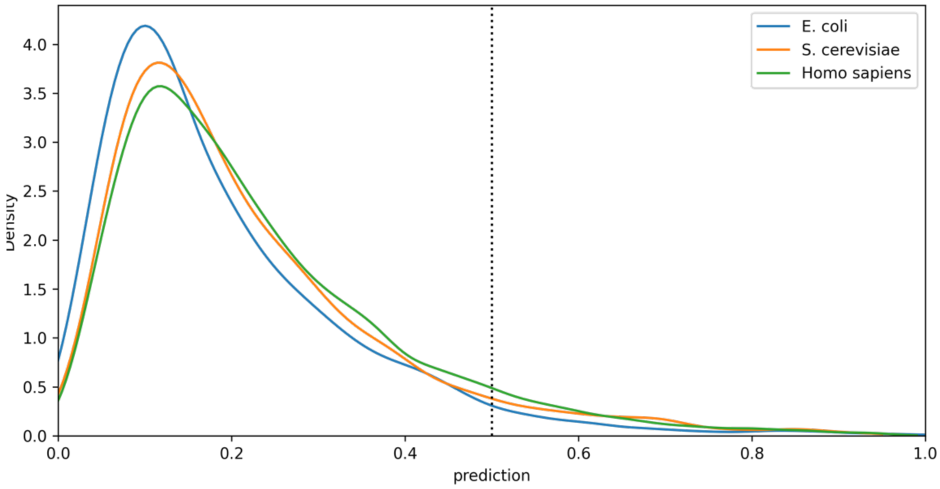

Fig. S6. The probability distribution function of SLiPP scores from the *E. coli*, yeast, and human proteomes. The dashed line indicates the hit threshold prediction score for SLiPP (0.5).

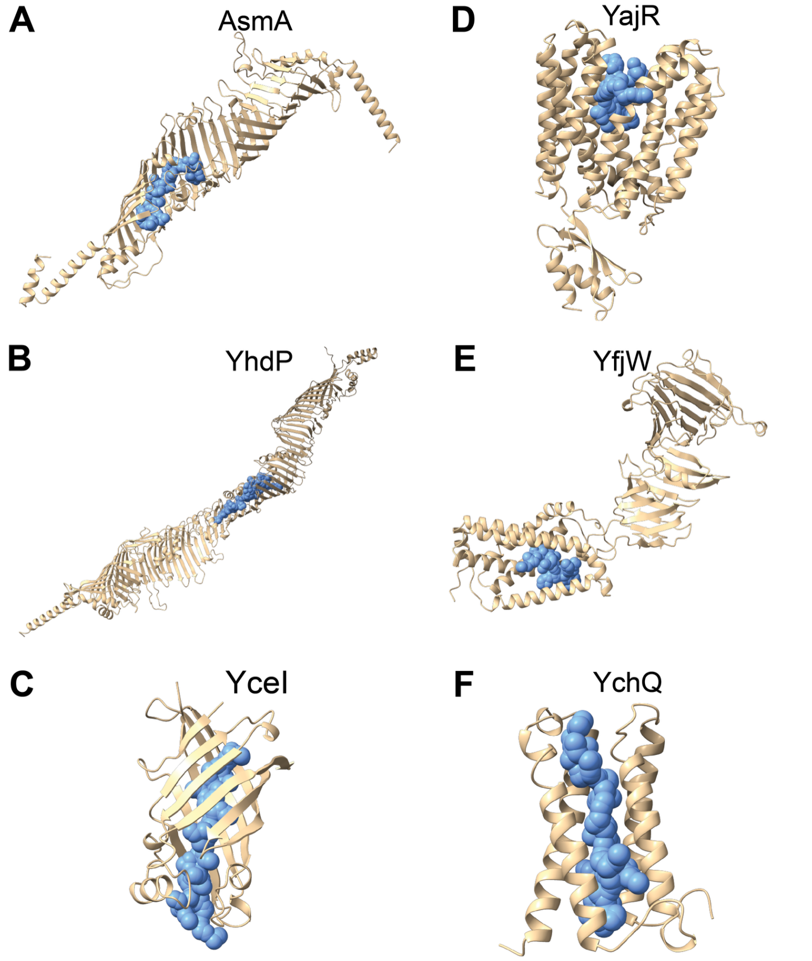

Fig. S7. SLiPP-predicted pockets (blue spheres) within the AlphaFold models of (A) AsmA (UniProt P28249), (B) YhdP (UniProt P46474), (C) YceI (UniProt P0A8X2), (D) YajR (UniProt P77726), (E) YfjW (UniProt P52138), and (F) YchQ (UniProt Q46755).

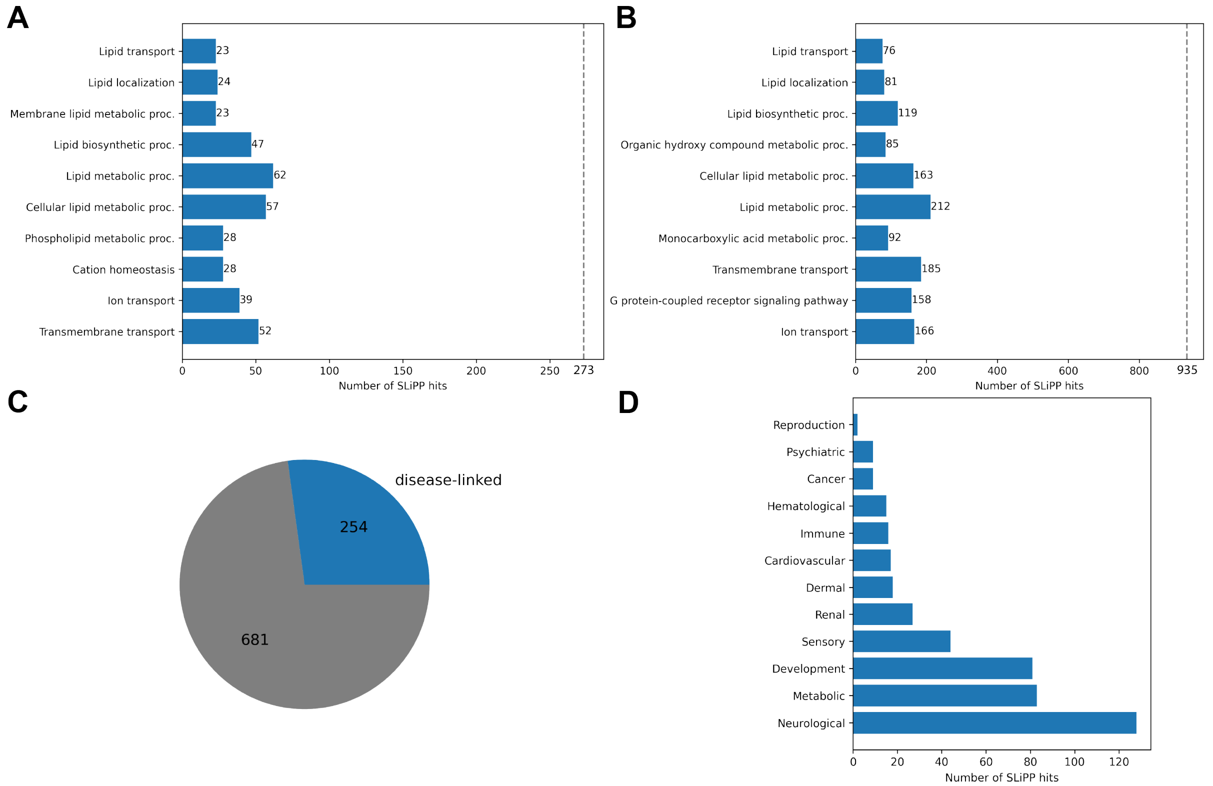

Fig. S8. SLiPP hits in the yeast and human proteome. (A, B) Numbers of SLiPP hits annotated with the corresponding GO terms in (A) yeast and (B) human proteome. The grey dashed line indicates the total number of SLiPP hits in the corresponding proteome. (C) Pie chart showing the proportion of SLiPP hits that are associated with one or more diseases in humans (as noted in the UniProt database). (D) Graph revealing the specific disease categories that SLiPP hits are associated with.

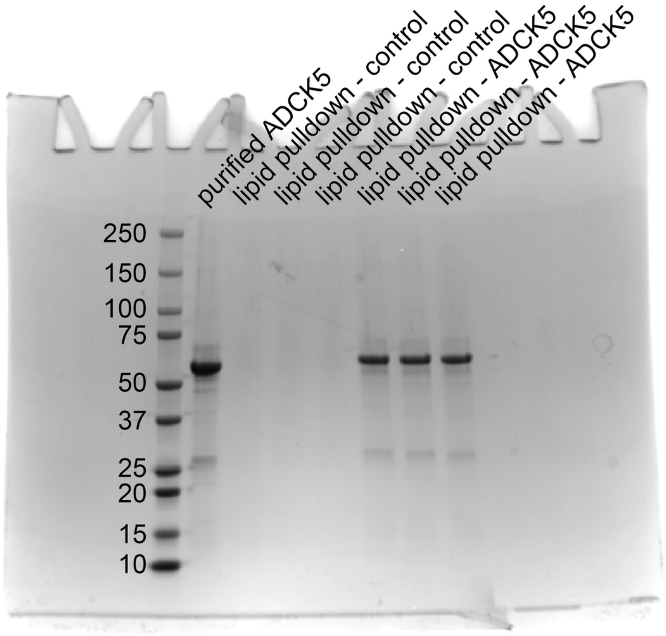

Fig. S9. SDS-PAGE analysis of purified ADCK5(68-580) and pulled-down sample for ligand identification by lipidomics. The gel was stained with Coomassie brilliant blue and the image was uncropped.

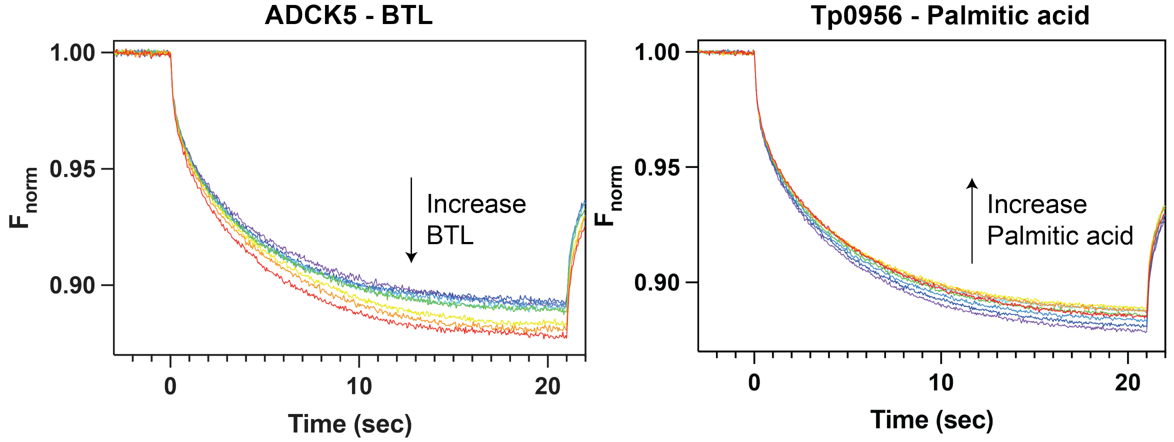

Fig. S10. MST time trace of fluorophore-labeled ADCK5 titrated with increasing concentration of BTL. 0 s indicates the time when IR laser was turned on. The fluorescence signal was normalized to the average fluorescence intensity from -1 to 0 second. In comparison, the MST time trace of fluorophore-labeled Tp0956 titrated with increasing concentration of palmitic acid on the right.

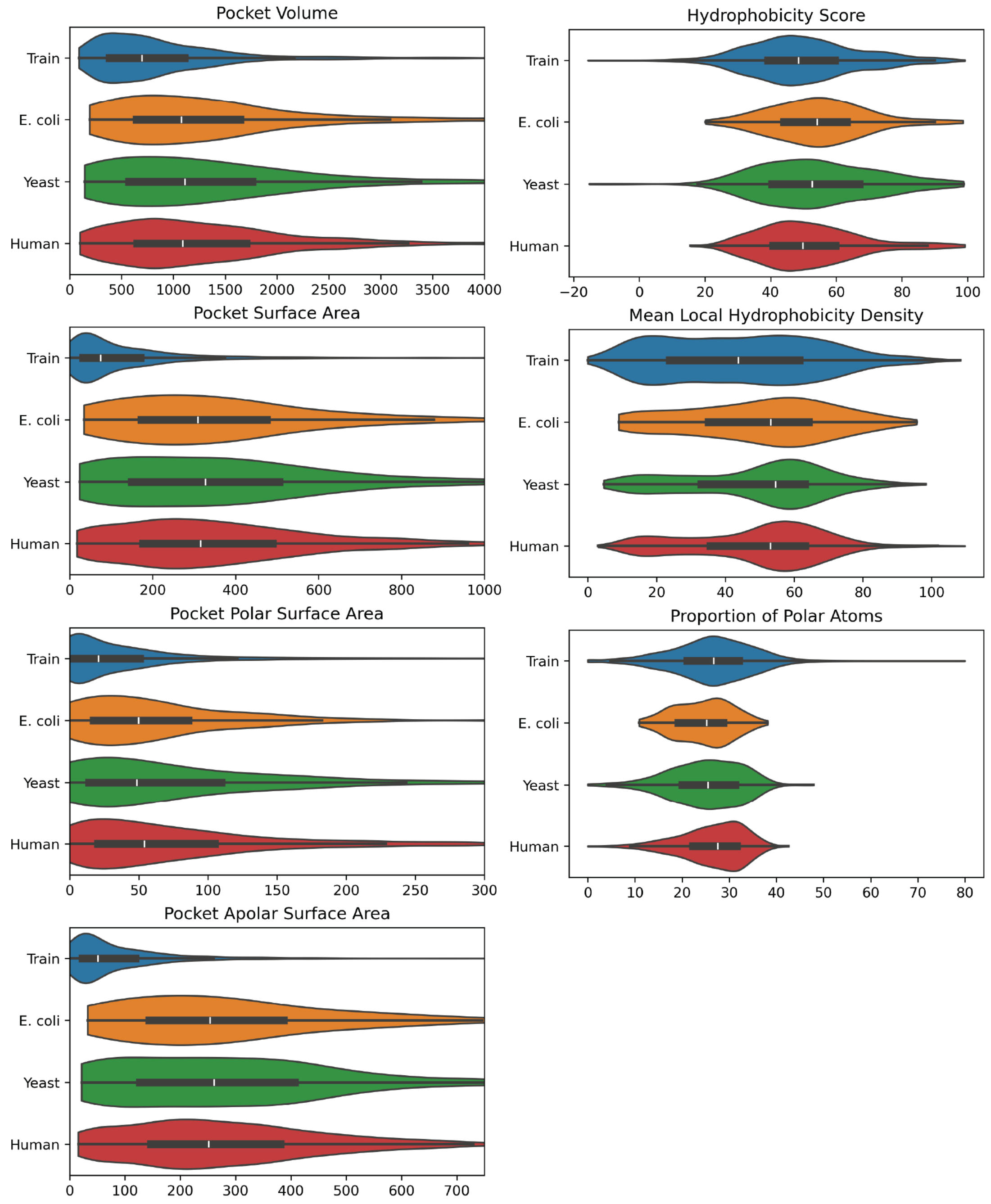

Fig. S11. Violin plots showing representative pocket descriptors of LBPs in the training dataset (train) and predicted LBPs in the *E. coli*, yeast, and human proteomes.

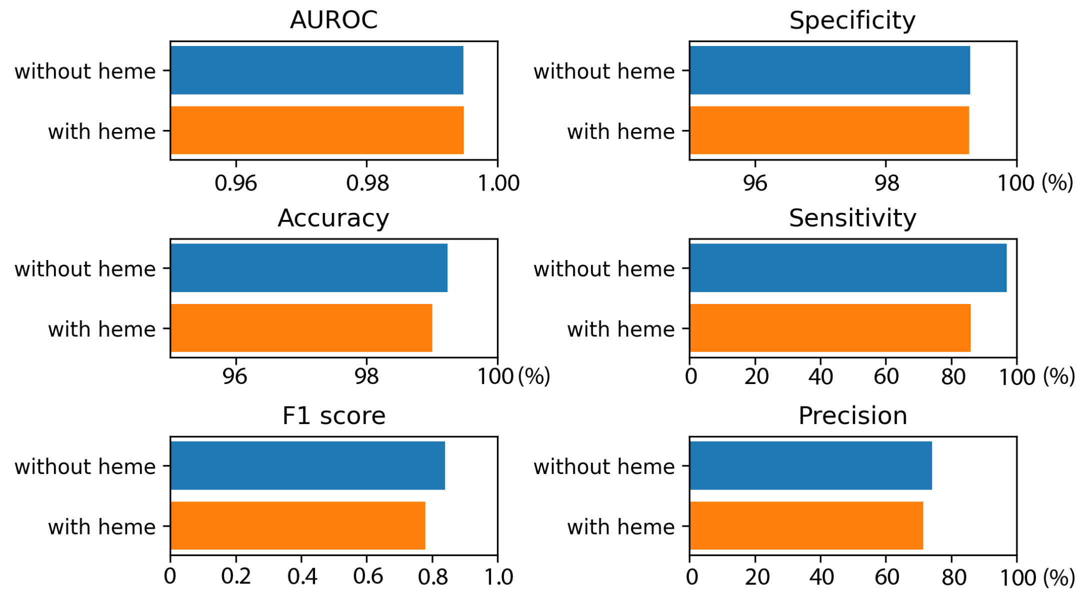

Fig. S12. Optimization of datasets with or without heme binding proteins. The performance of the classifier was assessed with a 10% independent test

Supporting tables

Table S1. Top 10 SLiPP hits of putative lipid binding proteins in *Borrelia burgdorferi* B31. Descriptions are annotations provided by UniProt.

GENE	DESCRIPTION
Lgt, BB_0362	Phosphatidylglycerol-prolipoprotein diacylglyceryl transferase
Lnt, BB_0237	Apolipoprotein N-acyltransferase
YajC, BB_0651	Sec translocon accessory complex subunit
BB_0584	Conserved hypothetical integral membrane protein
BB_0597	Methyl-accepting chemotaxis protein
BB_0368	Glycerol-3-phosphate dehydrogenase
BB_0117	UPF0073 membrane protein
BB_0747	Oligopeptide ABC transporter, permease protein
DnaJ, BB_0517	Chaperone protein
ResT, BB_B03	Telomere resolvase

Table S2. Top 10 SLiPP hits of putative lipid binding proteins in *Treponema pallidum pallidum*. Descriptions are annotations provided by UniProt.

GENE	DESCRIPTION
TP_0671	Sn-1,2-diacylglycerol cholinephosphotransferase
TP_0175	Uncharacterized protein
TP_0229	Type-4 uracil-DNA glycosylase
TP_0324	Uncharacterized protein
TP_0789	Uncharacterized protein
YidC, TP_0949	Membrane protein insertase
TP_0515	LptD C-terminal domain-containing protein
TP_0022	Uncharacterized protein
TP_0447	Uncharacterized protein
TP_0481	Uncharacterized protein

Table S3. Top 10 SLiPP hits of putative lipid binding proteins in *Chlamydia trachomatis*. Descriptions are annotations provided by UniProt.

GENE	DESCRIPTION
CT_850	Integral membrane protein
CydA, CT_013	Cytochrome Oxidase Subunit I
MenG, CT_428	Demethylmenaquinone methyltransferase
Lnt, CT_534	Apolipoprotein N-acyltransferase
CT_131	Possible Transmembrane Protein
CT_573	Uncharacterized protein
MraY, CT_757	Phospho-N-acetylmuramoyl-pentapeptide-transferase
UppS, CT_450	Isoprenyl transferase
Aas, CT_776	Acylglycerophosphoethanolamine Acyltransferase
BrnQ, CT_554	Amino Acid (Branched) Transport

Supporting files

File 1: Table of the PDB structures used to create the full dataset
File 2: Table of the apo PDB structures used to create the validation dataset
File 3: Table of the AlphaFold structures used to create the validation dataset
File 4: SLiPP hits in the *E. coli* proteome
File 5: SLiPP hits in the *S. cerevisiae* proteome
File 6: SLiPP hits in the *H. sapiens* proteome
File 7: Table of amino acids making up the putative lipid binding pocket of ADCK5
File 8: AlphaMissense analysis of ADCK5

References

1. Monera, O. D., T. J. Sereda, N. E. Zhou, C. M. Kay, and R. S. Hodges. 1995. Relationship of sidechain hydrophobicity and α-helical propensity on the stability of the single-stranded amphipathic α-helix. *J. Pept. Sci.* **1**: 319-329.
